# Case Report: An interesting case of vision and hearing loss revealing CNS recurrence in Philadelphia-positive acute lymphoblastic leukemia

**DOI:** 10.3389/fonc.2026.1758791

**Published:** 2026-05-04

**Authors:** Ifeoma Ike, Victor M. Samperio, Henry T. Tsai

**Affiliations:** 1 Internal Medicine, Eisenhower Health, Rancho Mirage, CA, United States; 2 Lucy Curci Cancer Center, Eisenhower Health, Rancho Mirage, CA, United States

**Keywords:** acute lymphoblastic leukemia, central nervous system, cerebrospinal fluid, dasatinib, magnetic resonance imaging, neuro-ophthalmologic, Philadelphia chromosome–positive, tyrosine kinase inhibitor

## Abstract

**Background:**

Central nervous system (CNS) relapse in acute lymphoblastic leukemia (ALL) may occur despite systemic remission and can present with isolated neuro-ophthalmologic symptoms. Early diagnosis may be challenging as the initial neuroimaging and ophthalmologic evaluation can be non-diagnostic.

**Case presentation/discussion:**

We report on a 57-year-old man with Philadelphia chromosome-positive (*Ph*+) B-cell ALL in remission on dasatinib maintenance therapy who presented with acute vision and hearing loss. The initial evaluation, including magnetic resonance imaging (MRI) and ophthalmologic examination, did not reveal a definitive etiology, and drug-induced toxicity was initially considered. However, cerebrospinal fluid (CSF) analysis demonstrated lymphoblasts, establishing the diagnosis of CNS relapse. Repeat imaging later revealed leptomeningeal and optic nerve sheath enhancement consistent with disease progression. The patient was treated with intrathecal chemotherapy, with subsequent clearance of CSF blasts.

**Conclusion:**

This case highlights delayed recognition of CNS relapse due to initial diagnostic anchoring and underscores the limitations of early imaging in detecting CNS involvement. CSF analysis remains the most sensitive diagnostic modality and should be pursued early in patients with ALL who present with unexplained visual or neurologic symptoms.

## Introduction

Acute lymphoblastic leukemia (ALL) is an aggressive hematologic malignancy characterized by clonal proliferation of lymphoid precursors that occurs more commonly in children than in adults (1). Central nervous system (CNS) involvement is present in a minority of patients at diagnosis, but remains a critical site of relapse, necessitating routine CNS-directed prophylaxis during treatment (1). Philadelphia chromosome-positive (*Ph*+) (*t*[9,22][q34;q11]) ALL represents a distinct subtype associated with historically poor prognosis, although outcomes have improved with the incorporation of tyrosine kinase inhibitors (TKIs) into treatment regimens (1).

CNS relapse of ALL may present with a wide spectrum of neurologic manifestations, including cranial neuropathies, headache, and visual disturbances (2). Importantly, early CNS involvement may not be detected on initial neuroimaging or ophthalmologic evaluation, particularly when the disease burden is low or confined to the leptomeninges or optic nerve (3). In such cases, cerebrospinal fluid (CSF) analysis remains the most sensitive diagnostic modality (2, 3).

We describe a patient with *Ph*+ B-cell ALL in remission on dasatinib maintenance therapy who developed vision and hearing loss. Although the initial evaluation suggested alternative etiologies, subsequent evaluation revealed CNS relapse as the underlying cause. This case highlights the importance of early CSF evaluation in patients with ALL presenting with neuro-ophthalmologic symptoms.

## Clinical course

A 57-year-old man with a medical history of hypertension, hyperlipidemia, and *Ph*+ B-cell ALL involving a T9 vertebral mass presented to the emergency department in the fourth week of July 2025 with visual and auditory disturbances. The patient had initially been diagnosed with *Ph*+ B-cell ALL in April 2022 and was treated with eight cycles of rituximab plus hyper-cyclophosphamide/vincristine/doxorubicin/dexamethasone (R-hyper-CVAD) with intrathecal methotrexate and cytarabine (MA). He continued on dasatinib maintenance therapy (initially started on May 17, 2022). At the time of diagnosis, he was evaluated by a bone marrow transplant specialist who determined that bone marrow transplantation was not indicated. The patient achieved remission in June 2023 (**Table 1**).

**Table 1:** Clinical course timeline highlighting significant events and relevant additional information.

Timeline	Clinical event	Additional information
Early April 2022	Diagnosed with B-cell ALL after FNA of T9 vertebral mass and peripheral blood flow cytometry showed B-cell ALL. Bone marrow biopsy showed 100% replacement of B-cell lymphoblastic leukemia, with cytogenetics showing *Ph*+ and FISH showing translocation 9–22 positive.	CSF flow cytology negative
Late April 2022	R-Hyper-CVAD/MA cycle 1a started. Dasatinib started after cycle 1b (May 17, 2022).	Referred to the local tertiary center for bone marrow transplant evaluation
June 2023	Eight cycles of R-Hyper-CVAD/MA completed and now only on dasatinib maintenance. BCR-ABL P210, P190 negative	Treatment course intermittently interrupted due to hospitalizations for recurrent viral infections and pancytopenia
August 2023–June 2025	Multiple follow-up visits to review flow cytometry and repeat BCR-ABL. Flow cytometry and BCR-ABL remain negative.	Two hospitalizations during this time for viral infections and pancytopenia. Chronic acyclovir started during this time
Early July 2025 (July 1, 2025)	Acute left-sided vision loss. Brain MRI negative. Dasatinib discontinued	Evaluated by ophthalmology who recommended stopping dasatinib therapy.
Late July 2025–Mid August 2025	Admission for persistent left-sided vision loss and right-sided blurry vision. Lumbar puncture performed August 13, 2025	MRI brain without contrast showed tiny left cerebellar lacunar infarct. CSF flow cytometry showed B lymphoblast population.
Mid-August 2025	Transferred to a tertiary care center for further evaluation and treatment. Repeat MRI at the new facility showed leptomeningeal and optic nerve sheath enhancement.	Bone marrow biopsy showed evidence of recurrent B-ALL.
Mid-August–Early September 2025	Intrathecal MA course started and completed. Left-sided vision loss stable. Right-sided vision loss improved. Dasatinib re-started during this time.	Ophthalmology at the tertiary center determined no ophthalmic arterial event occurred.
September 2025	Hospitalization at the tertiary center concluded with discussion for transfer to an alternative tertiary center for CAR-T therapy.	CSF analysis negative for blast cells

ALL, acute lymphoblastic leukemia; CAR-T, chimeric antigen receptor T-cell therapy; CSF, cerebrospinal fluid; FNA, fine-needle aspiration: FISH, fluorescence in situ hybridization; MRI, magnetic resonance imaging; MA, methotrexate/cytarabine; Ph+, Philadelphia chromosome; R-Hyper-CVAD, rituximab plus hyper-cyclophosphamide/vincristine/doxorubicin/dexamethasone.

In the first week of July 2025, the patient developed acute vision loss in his left eye. He presented to an outside emergency department where magnetic resonance imaging (MRI) of the brain revealed no acute intracranial abnormalities. He was subsequently evaluated by two ophthalmologists. Visual acuity was 20/20 in the right eye, and there was absence of light perception in the left eye. Intraocular pressure, anterior segment examination, fundus examination, and external ocular findings were otherwise normal. The vision loss was initially suspected to be permanent and potentially related to dasatinib therapy. In response, his oncologist discontinued dasatinib in the third week of July 2025 and recommended further evaluation by a retina specialist; however, this follow-up was not completed.

The morning of his current presentation in the fourth week of July 2025, the patient noted acute-onset blurry vision in his right eye while exercising. He also reported progressive bilateral hearing loss over several weeks, which was worse on the left side. This was accompanied by a daily right-sided throbbing temporal headache for 1 month, occasionally radiating to the contralateral temporal region and partially relieved by acetaminophen. He denied fever, chills, fatigue, chest pain, dyspnea, abdominal pain, gastrointestinal symptoms, or additional neurologic deficits. His medications were losartan, rosuvastatin, aspirin, and a multivitamin. Family history was notable for uterine cancer in his mother.

On presentation, vital signs were stable and neurologic examination was unremarkable aside from visual and auditory deficits. Laboratory evaluation showed hemoglobin of 13.5 g/dl and platelets of 146 × 10^9^/L, consistent with his baseline values. Troponin, international normalized ratio, urinalysis, urine drug screen, and respiratory viral testing were unremarkable. Electrocardiogram demonstrated normal sinus rhythm. Chest radiography and computed tomography of the head showed no acute abnormalities.

MRI of the brain demonstrated a tiny left cerebellar lacunar infarct and nonspecific white matter changes. MRI of the orbits was unremarkable. Ophthalmologic examination revealed visual acuity of 20/100 in the right eye and no light perception in the left eye. A left afferent pupillary defect was present. Dilated fundus examination showed a normal optic nerve, macula, and retinal vasculature in the right eye, while the left optic nerve appeared slightly pale with otherwise normal retinal findings, which contrasted the initial left optic nerve findings before admission.

Given the patient’s history of *Ph*+ ALL, concern for relapse prompted oncology consultation. A lumbar puncture was recommended, which demonstrated lymphoblasts on cytology. CSF flow cytometry revealed an abnormal B-lymphoblast population (28%) expressing CD34, CD19, CD22, CD10, and human leukocyte antigen-DR (HLA-DR). Peripheral blood flow cytometry obtained during the hospitalization (August 13, 2025) showed no evidence of circulating blasts.

As dasatinib has been rarely associated with non-arteritic anterior ischemic optic neuropathy, the patient’s vision loss was initially attributed to dasatinib-associated ocular and auditory toxicity. However, the presence of blasts in the CSF confirmed CNS relapse of ALL as the likely etiology of his vision and hearing loss.

ABL1 kinase domain mutation testing was performed, and the results were negative. The treatment options under consideration included enrollment in a clinical trial, blinatumomab with or without a TKI, inotuzumab ozogamicin, chimeric antigen receptor T-cell (CAR-T) therapy, or other salvage chemotherapy. As some of these therapies were not available locally, plans were made to re-engage his prior transplant physician for potential transfer and advanced treatment options.

After transfer to a tertiary care center, repeat contrast-enhanced MRI of the brain and orbits demonstrated diffuse leptomeningeal enhancement within the posterior fossa and bilateral optic nerve sheath enhancement. Bone marrow biopsy performed on August 18, 2025, revealed 2.5% aberrant B lymphoblasts, consistent with recurrent B-cell ALL. The patient received intrathecal administration of MA (two doses of each total) between August 19 and September 2, 2025. Serial CSF analyses demonstrated clearance of blasts after therapy, and treatment was stopped on September 2, 2025. Ophthalmology was consulted and determined that no ophthalmic arterial event occurred. Dasatinib was resumed. Toward the end of the patient’s hospitalization, his left-sided vision loss was stable and his left-sided hearing loss improved. His right-sided vision loss improved back to his baseline. Transfer to an alternative tertiary center was planned for further treatment of craniospinal radiation therapy (XRT) *versus* focused XRT aimed at optic nerves, then CAR-T infusion after XRT is complete.

## Discussion

CNS relapse in ALL represents a clinically significant complication that may occur despite systemic remission and ongoing therapy (1). Although CNS involvement is relatively uncommon at the initial diagnosis, it remains a well-recognized site of relapse and may present with a range of neurologic manifestations, including cranial neuropathies, headache, and visual disturbances (2, 4). In some cases, neuro-ophthalmologic symptoms may be the initial or predominant manifestation of CNS disease (2).

Optic neuropathy may occur through leukemic infiltration of the optic nerve, compression from leptomeningeal disease, or ischemic injury related to leukemic infiltration of the surrounding structures (2). Similarly, auditory symptoms may result from the involvement of cranial nerve VIII or adjacent leptomeningeal structures (5). These mechanisms can produce progressive visual and auditory deficits, as observed in our patient. Early optic nerve involvement may present as retrobulbar optic neuropathy, in which funduscopic examination is initially normal (5). Objective ophthalmologic measures such as formal visual field testing, optical coherence tomography, or visual evoked potentials were not available in this case, which limited further characterization of the optic neuropathy. However, the presence of an afferent pupillary defect and later development of optic disc pallor reflect the progression of optic nerve injury (2).

**Figure 1:**
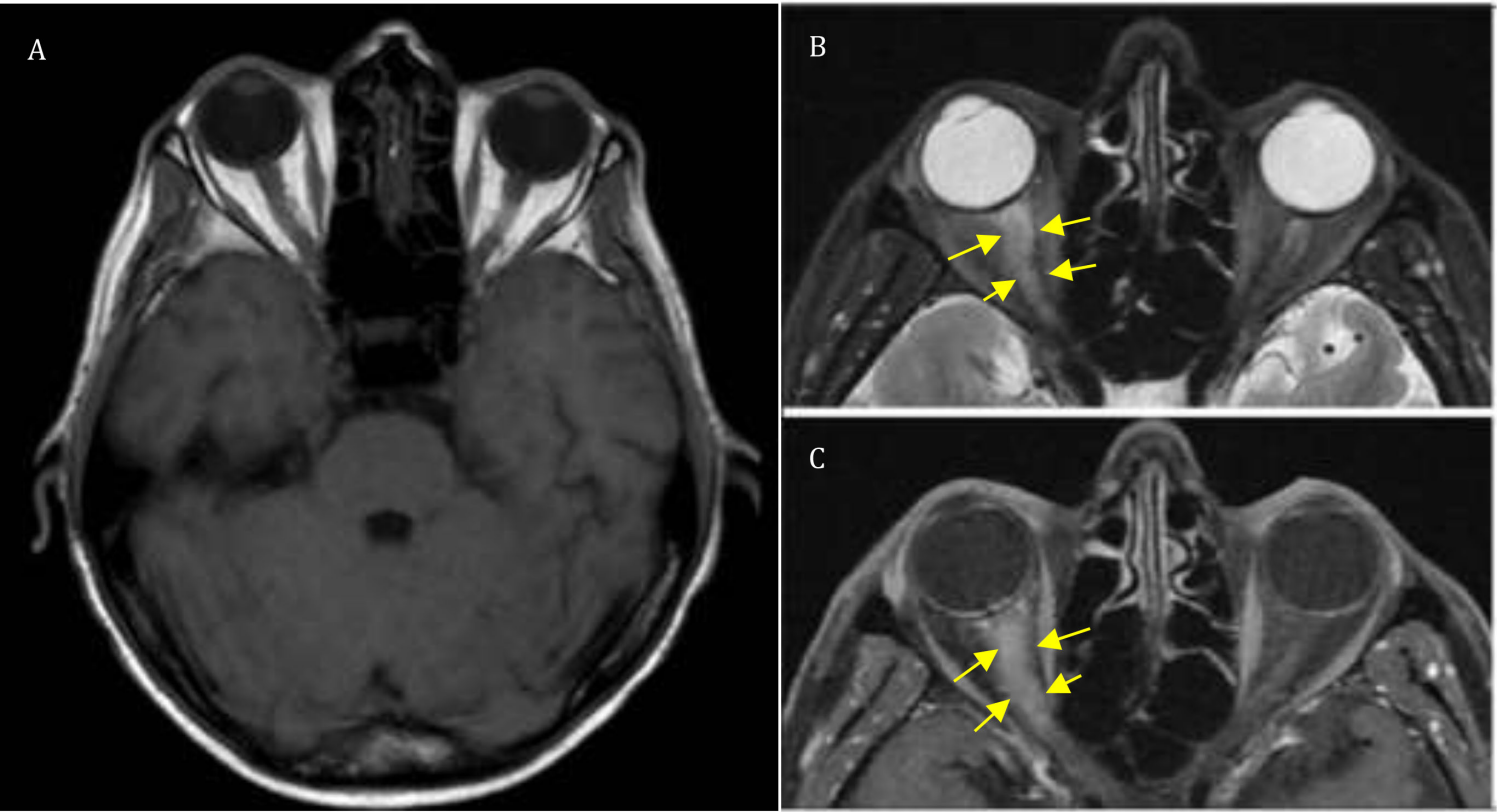
Two different MRI brain images comparing optic nerve enlargement. **(A)** Patient’s initial brain MRI without optic nerve thickening. **(B, C)** Images showing right optic nerve enlargement (referenced from the Indian Journal of Ophthalmology—Case Reports 3(3):873–876, Jul–Sep 2023). *Yellow arrows* indicate the areas of enlargement.

This case also highlights the limitations of neuroimaging in early CNS relapse. Previous reports demonstrated that MRI may initially appear normal or demonstrate subtle findings, such as optic nerve thickening or nonspecific enhancement, particularly in the early stages of CNS involvement (**Figure 1**) (6).

This may reflect low disease burden or early leptomeningeal involvement not yet detectable radiographically (3). The subsequent development of leptomeningeal and optic nerve sheath enhancement on repeat imaging is consistent with disease progression and increased leukemic infiltration (3). This evolution indicates that a normal initial MRI does not exclude CNS involvement. Given these limitations, a structured diagnostic approach is essential. In patients with a history of leukemia presenting with acute or subacute visual symptoms, the initial evaluation should include ophthalmologic examination and neuroimaging; however, non-diagnostic findings should not delay further investigation. CSF analysis remains the most sensitive diagnostic modality for detecting CNS relapse of ALL (3). CSF cytology and flow cytometry can identify leukemic blasts even when peripheral blood studies and imaging are non-diagnostic (2, 3). In the present case, the initial neuroimaging did not demonstrate definitive CNS leukemic involvement; however, lumbar puncture revealed a population of abnormal B lymphoblasts, consistent with CNS relapse. These findings emphasize the critical role of early lumbar puncture in patients with unexplained neuro-ophthalmologic symptoms and a history of ALL.

Dasatinib-associated optic neuropathy was considered in the differential diagnosis. Dasatinib binds to and inhibits the growth-promoting activities of BCR–ABL tyrosine kinase and is commonly used in the treatment of *Ph*+ ALL (7). Rare cases of optic neuropathy associated with dasatinib have been described in the literature (4, 8, 9). In Monge et al., the patient had loss of visual acuity in his right eye, as well as bilateral visual field involvement after dasatinib use (8). In these cases, patients typically experienced improvement in visual symptoms following discontinuation of the drug (2) (**Table 2**). In contrast, our patient experienced persistent vision loss despite cessation of dasatinib, further supporting CNS relapse rather than drug-induced optic neuropathy.

**Table 2:** Summarization of key differences between differentials for optic neuropathy.

Feature	Dasatinib-induced optic neuropathy	Optic neuropathy from CNS leukemia relapse	Vascular optic neuropathy (ischemic/stroke-related)
Typical onset	Subacute; usually weeks to months after starting dasatinib (8)	Can occur at relapse; may occur despite bone marrow remission (3)	Sudden onset (minutes–hours) (10)
Laterality	Often bilateral, but asymmetric (8)	Often bilateral, although may start unilateral (11)	Usually unilateral in anterior ischemic optic neuropathy; may be homonymous defects if occipital stroke (5)
Associated symptoms	Gradual visual field defects; may have mild headache; usually no systemic neurologic deficits (8)	Headache, nausea, vomiting, cranial nerve deficits, meningismus, or other CNS symptoms (2)	Acute neurologic deficits (aphasia, hemiparesis) if stroke; vascular risk factors common (10)
Fundoscopic findings	Mild optic disc edema or pallor; sometimes normal early (6)	Optic disc edema, optic nerve swelling, or infiltrative appearance (3)	Disc edema in non-arteritic anterior ischemic optic neuropathy (5)
Visual field pattern	Often arcuate or paracentral scotomas (8)	Variable; severe vision loss common (11)	Altitudinal field defect (AION) or homonymous defects (posterior stroke) (10)
MRI brain/orbit	Often normal or nonspecific optic nerve thickening without mass or leptomeningeal disease (8)	Optic nerve enhancement, thickening, or leptomeningeal involvement; other CNS lesions may be present (3)	Infarct in optic pathways (optic nerve, chiasm, optic tract, or occipital cortex) (5)
CSF findings	Lack evidence of malignant cells present in the CSF (8)	Frequently malignant cells/blasts present in the CSF (3)	Usually normal, unless secondary hemorrhage (5)
Prognosis	Often partially reversible if drug stopped early (2, 8)	Often poor visual recovery; associated with leukemia progression (11)	Variable; vision often permanently impaired (12)

AION, anterior ischemic optic neuropathy; CSF, cerebrospinal fluid; CNS, central nervous system.

The possibility of relapse despite ongoing dasatinib therapy raises additional clinical considerations. Relapse during dasatinib therapy in *Ph*+ ALL is most often driven by BCR–ABL1 kinase domain mutations, particularly T315I, which confers broad resistance to second-generation TKIs (13, 14). However, an ABL1 kinase domain mutation including T315I was not identified in our patient. Other risk factors include adverse genetic profiles such as *IKZF1*-plus lesions, which predict inferior survival (15). Ponatinib demonstrates activity against both wild-type and resistant BCR–ABL1, and clinical studies show superior molecular response and survival outcomes compared with earlier-generation TKIs (16). Combination strategies with blinatumomab, inotuzumab ozogamicin, CAR-T cell therapy, or stem cell transplantation are increasingly employed in relapsed or refractory disease (16, 17).

## Conclusion

Overall, this case illustrates that CNS relapse of *Ph*+ ALL may present with isolated neuro-ophthalmologic symptoms and minimal initial imaging findings. Delayed recognition may occur due to diagnostic anchoring on more common or treatment-related etiologies. Prompt CSF analysis is essential for establishing the diagnosis and initiating appropriate therapy and should be strongly considered in patients with leukemia who present with unexplained visual or neurologic symptoms.

## Data Availability

The original contributions presented in the study are included in the article/supplementary material. Further inquiries can be directed to the corresponding author.

## References

[1] Terwilliger T Abdul-Hay M . Acute lymphoblastic leukemia: a comprehensive review and 2017 update - PubMed. Blood Cancer J. (2017) 7. 10.1038/bcj.2017.53 PMC5520400 28665419

[2] Jo S Yoo JW Kim S Lee JW Im SA Cho B . Case report: First report of isolated central nervous system lymphoblastic crisis in a child with chronic myeloid leukemia on dasatinib therapy - PubMed. Front Oncol. (2023) 13. 10.3389/fonc.2023.1122714 PMC10076740 37035148

[3] Lee V Farooq AV Shah HA . Leukemic and lymphomatous optic neuropathy: a case series. J Neuro-Ophthalmol. (2021) 41. 10.1097/WNO.0000000000001365 34629409

[4] Faderl S O'Brien S Pui CH Stock W Wetzler M Hoelzer D . Adult acute lymphoblastic leukemia: concepts and strategies - PubMed. Cancer. (2010) 116. 10.1002/cncr.24862 PMC5345568 20101737

[5] Bellows D . Neuro-ophthalmology illustrated, 3rd edition: by valérie biousse and nancy J. Newman, new york, NY, thieme medical publishers, inc. 2020, 696 pp. 662 illustrations, 69 video clips, $119.99 (softcover), ISBN: 9781684200740, (e-book) ISBN: 9781684200757. Neuro-Ophthalmology. (2020) 44.

[6] Barigali A Pai M Ganesh S . Anti-MOG antibody optic neuritis in AML – A case report. Indian J Ophthalmol - Case Rep. (2023) 3.

[7] Korashy HM Rahman AF Kassem MG . Dasatinib - pubmed. Profiles Drug Substances Excipients Related Method. (2014) 39. 10.1016/B978-0-12-800173-8.00004-0 24794907

[8] Monge KS Gálvez-Ruiz A Alvárez-Carrón A Quijada C Matheu A . Optic neuropathy secondary to dasatinib in the treatment of a chronic myeloid leukemia case. Saudi J Ophthalmol. (2015) 29. 10.1016/j.sjopt.2014.12.004 PMC4487962 26155085

[9] Kurtz JE Andrés E Veillon F Maloisel F Gentine A Herbrecht R . Hearing loss due to acute leukemia - PubMed. Am J Med. (2000) 109. 10.1016/s0002-9343(00)00563-5 11184771

[10] Hayreh SS . Ischemic optic neuropathy - PubMed. Prog Retinal Eye Res. (2009) 28. 10.1016/j.preteyeres.2008.11.002 19063989

[11] Chen CY Jhang JP Lai YJ Lin CW Chou ET Lin CC . Clinical manifestations and ophthalmic outcomes of leukemic retinopathy and optic neuropathy in patients with acute leukemia. Invest Ophthalmol Visual Sci. (2025) 66. 10.1167/iovs.66.11.2 PMC12320904 40747974

[12] Miller NR Arnold AC . Current concepts in the diagnosis, pathogenesis and management of nonarteritic anterior ischaemic optic neuropathy. Eye. (2014) 29. 10.1038/eye.2014.144 PMC4289822 24993324

[13] Ravandi F O'Brien SM Cortes JE Thomas DM Garris R Faderl S . Long-term follow-up of a phase 2 study of chemotherapy plus dasatinib for the initial treatment of patients with Philadelphia chromosome-positive acute lymphoblastic leukemia - PubMed. Cancer. (2015) 121. 10.1002/cncr.29646 PMC4666803 26308885

[14] Foà R . Ph-Positive acute lymphoblastic leukemia - 25 years of progress - PubMed. N Engl J Med. (2025) 392. 10.1056/NEJMra2405573 40367376

[15] Jabbour E Haddad FG Short NJ Kantarjian H . Treatment of adults with Philadelphia chromosome-positive acute lymphoblastic leukemia-from intensive chemotherapy combinations to chemotherapy-free regimens: a review - PubMed. JAMA Oncol. (2022) 8. 10.1001/jamaoncol.2022.2398 35834222

[16] Raza MZ Khwaja HF Arshad HME Zulnorain Maqsood M Nadeem AA . Comparison of third-generation tyrosine kinase inhibitor (TKI) ponatinib with first- and second-generation TKIs for treatment of Philadelphia chromosome-positive acute lymphoblastic leukemia: a systematic review and bias-corrected meta-analysis - PubMed. Crit Rev Oncology/Hematol. (2025) 213. 10.1016/j.critrevonc.2025.104806 40517974

[17] Dini G Capolsini I Cerri C Massei MS Mastrodicasa E Perruccio K . Acute lymphoblastic leukemia relapse presenting with optic nerve infiltration - PubMed. SAGE Open Med Case Rep. (2023) 11. 10.1177/2050313X231175020 PMC10214062 37250823

